# Identification of candidate genes from androgenic gland in *Macrobrachium nipponense* regulated by eyestalk ablation

**DOI:** 10.1038/s41598-021-99022-4

**Published:** 2021-10-06

**Authors:** Shubo Jin, Yin Fu, Yuning Hu, Hongtuo Fu, Sufei Jiang, Yiwei Xiong, Hui Qiao, Wenyi Zhang, Yongsheng Gong, Yan Wu

**Affiliations:** 1 Key Laboratory of Freshwater Fisheries and Germplasm Resources Utilization, Ministry of Agriculture, Freshwater Fisheries Research Center, Chinese Academy of Fishery Sciences, 9 Shanshui East Road, Wuxi, 214081 Jiangsu Province People’s Republic of China; 2 Key Laboratory of Marine and Estuarine Fisheries, Ministry of Agriculture, East China Sea Fisheries Research Institute, Chinese Academy of Fishery Sciences, Shanghai, 200090 People’s Republic of China; 3 Wuxi Fisheries College, Nanjing Agricultural University, Wuxi, 214081 People’s Republic of China

**Keywords:** Genetics, Molecular biology, Zoology

## Abstract

The eyestalk of crustaceans, such as *Macrobrachium nipponense*, contains many neurosecretory hormones affecting the process of reproduction, molting, metabolism of glucose, and other functions. In this study, important metabolic pathways and candidate genes involved in male sexual development were selected from *M. nipponense*. The methodology involved performing long-read and next generation transcriptome sequencing of genes from the androgenic gland after eyestalk ablation. qPCR analysis revealed that the mRNA expression of *Mn-IAG* was significantly increased after ablation of both the single-side (SS) and double-side (DS) eyestalk, compared with the control group (CG). The long-read transcriptome generated 49,840 non-redundant transcripts. A total of 1319, 2092 and 4351 differentially expressed genes (DEGs) were identified between CG versus SS, SS versus DS and CG versus DS, respectively. These data indicated that ablation of the double-sided eyestalk played stronger regulatory roles than the single-side ablation on male sexual development in *M. nipponense*. This was consistent with the qPCR analysis. Cell Cycle, Cellular Senescence, Oxidative Phosphorylation, Glycolysis/Gluconeogenesis and Steroid Hormone Biosynthesis were the primary enriched metabolic pathways in all three comparisons, and the important genes from these metabolic pathways were also selected. qPCR permitted secondary confirmation of ten DEGs identified through RNA-seq. RNAi-mediated silencing analyses of Hydroxysteroid dehydrogenase like 1 (*HSDL1*) revealed that *HSDL1* has a positive regulatory effect on testes development. This study provides valuable insight into male sexual development in *M*. *nipponense*, including metabolic pathways and genes, paving the way for advanced studies on male sexual development in this species and in other crustaceans.

## Introduction

The oriental river prawn, *Macrobrachium nipponense* (Crustacea; Decapoda; Palaemonidae), is widely distributed in China and other Asian countries^1–3^. This species is commercially important with an annual aquaculture production of ~ 205,010 tons in 2016^4^. Similar to other *Macrobrachium* species, male prawns grow faster and reach a larger size at harvest time^2^. Thus, male prawns are preferred in the *M*. *nipponense* aquaculture industry. In addition, the rapid development of testes in the reproductive season is another issue restricting the sustainable development of *M*. *nipponense*. Previous studies revealed that the testes of *M*. *nipponense* reach sexual maturity within 40 days after hatching^5^. Thus, inbreeding will occur between the newborn prawns. Inbreeding will lead to a decrease in resistance to adversity in their offspring, small scale of market prawn, and a degradation in germplasm resources. Therefore, it is important to fully understand the mechanism of male sexual differentiation and development with the aim of producing all-male progeny on a commercial scale. This can be accomplished by regulating the process of testes development in *M*. *nipponense*.

The androgenic gland is a unique tissue in crustacean species. It has been shown to play an essential role in male sexual differentiation and development in crustacean species. Many studies have reported that the androgenic gland and its secreted hormones promote the driving of male sexual differentiation, the establishment of male sexual characteristics and the development of the testes in crustacean species^6,7^. For example, the ablation of the androgenic gland from male *M. rosenbergii* resulted in the sex reversal; a phenomenon that has been termed “neo-female”^6,7^. The insulin-like androgenic gland hormone (*IAG*) is an important hormone secreted by the androgenic gland. IAG was shown to promote male sexual differentiation and development in many crustacean species^8–10^. It is the most important male sex-related gene acknowledged in many crustacean species. Knockdown of *IAG* expression by RNAi in male *M*. *rosenbergii* can also result in sex reversal^11^. *IAG*, specially expressed in the androgenic gland of *M*. *nipponense* and other crustaceans, was also implicated in the maintenance of male secondary sex characteristics, spermatogenesis, reproductive strategies and the regulation of growth^8–10,12–15^. Recent studies on the androgenic gland have become more prominent in the literature. A series of transcriptomes from the androgenic gland have been constructed in *M*. *nipponense*^16–18^, and a series of important genes from the androgenic gland have been implicated as having an essential role in male sexual development^19–22^. In addition, the histological observations during different post-larval developmental stages indicated that the development of the androgenic gland has regulatory roles in the development of testes^5^.

The eyestalk of crustacean species has many neurosecretory hormones. The X-organ–SG complex (XO–SG) was identified as a principal neuroendocrine gland located in the eyestalk^23^. It stores and releases the crustacean hyperglycemic hormone (*CHH*) superfamily of neurohormones, including *CHH*, ion transport peptides (*ITP*), gonad-inhibiting hormone (*GIH*), molt inhibiting hormone (*MIH*) and mandibular organ-inhibiting hormone (*MOIH*), playing essential roles in reproduction^24–26^, molting^27–29^, metabolism of glucose^30,31^ and other function^32–34^. Knockdown of *GIH* by RNAi promoted ovarian development in *M*. *nipponense*^35^. Knockdown of *MIH* by RNAi promoted molting in *M*. *nipponense*^36^. *CHH* has been shown to promote testes development in *M*. *nipponense*^37^.

In this study, the vital metabolic pathways and genes involved in male sexual differentiation and development in *M*. *nipponense* were selected by performing long-read and next generation transcriptome profiling analysis of the androgenic gland after the ablation of the single-side and double-side eyestalk. The functions of Hydroxysteroid dehydrogenase like 1 (*HSDL1*), which was predicted to be involved in the mechanism of male sexual development in this study and in a previous study^38^, were further analyzed in depth by using qPCR analysis and RNAi. This study provides valuable evidence of male sexual differentiation and development in *M*. *nipponense*, as well as in other crustacean species.

## Results

### The expression analysis of *Mn-IAG* after eyestalk ablation

**Figure 1:**
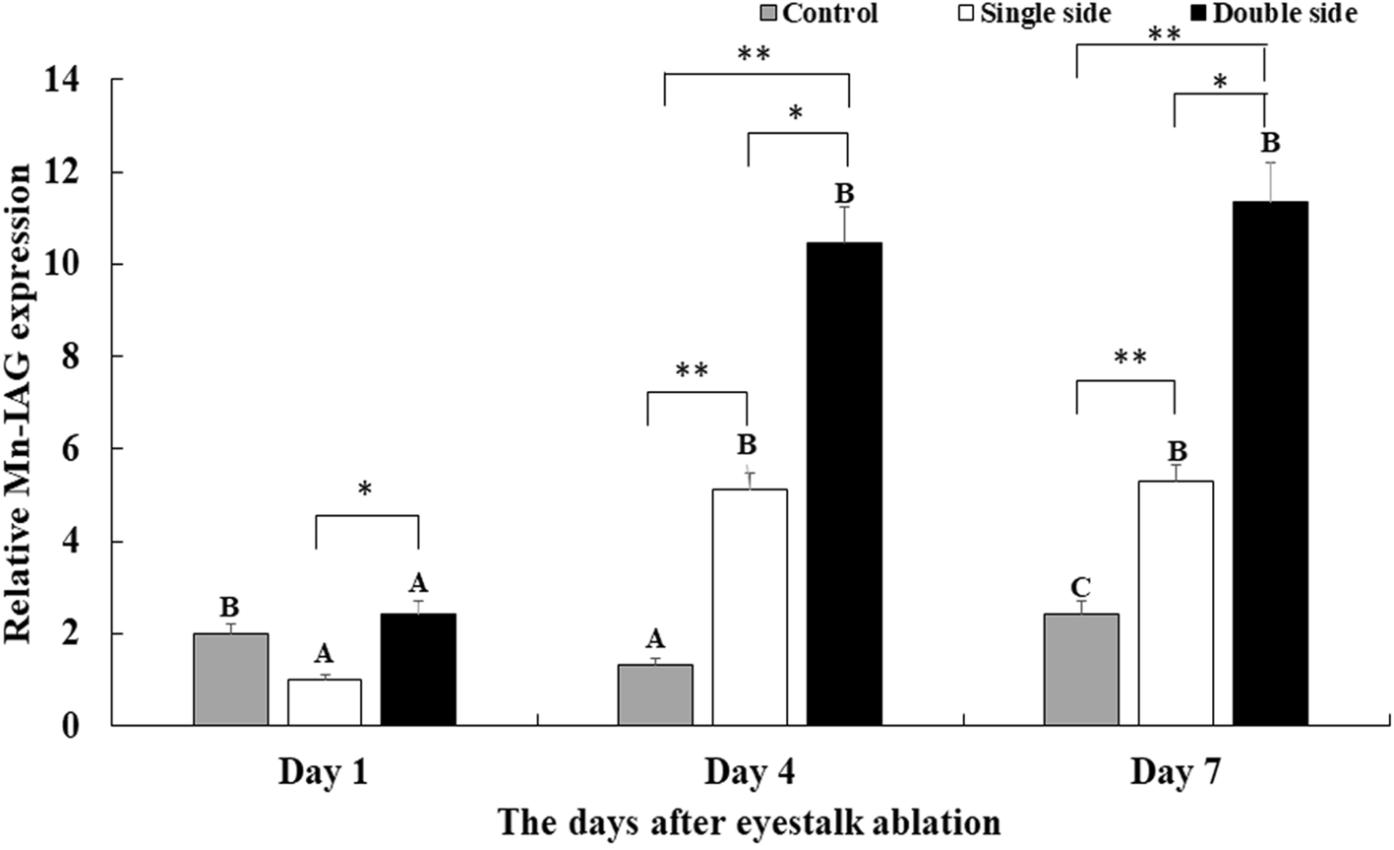
Measurement of the expression of *Mn-IAG* after the ablation of eyestalk. The amount of *Mn-IAG* mRNA was normalized to the *EIF* (eukaryotic translation initiation factor 5A) transcript level. Data are shown as mean ± SD (standard deviation) of tissues from three separate individuals*.* Capital letters indicated expression difference between different days in the same group. * (*P* < 0.05) and ** (*P* < 0.01) indicates significant expression difference between different groups at the sample day.

The mRNA expression of *Mn-IAG* was measured in three groups: control group (CG), single-side ablation of eyestalk (SS), and double-side ablation of eyestalk (DS) (Fig. 1). The data showed that the mRNA expression of *Mn-IAG* increased with the time of eyestalk ablation in the SS group and the DS group. The mRNA expression of *Mn-IAG* was ~ 5-folder higher at day 4 and day 7 than for day 1 in both the SS and DS groups (*P* < 0.01). However, *Mn-IAG* expression was only slightly higher at day 7 than day 4 in both the SS and DS groups, showing no significant difference (*P* > 0.05). *Mn-IAG* expression in the DS group was ~ 2-folder higher than that in the SS group on the same day and showed significant difference (*P* < 0.05).

### Long-read transcriptome

A total of 22.83 GBs of clean data were generated in the long-read transcriptome. A total of 160,496 high-quality transcripts were obtained with a mean length of 2230 bp. Finally, 49,840 non-redundant transcripts were identified in the long-read transcriptome. These unigenes were annotated, based on the *M. nipponense* genome, and a total of 43,115 unigenes matched known sequences in the *M. nipponense* genome. All of the non-redundant transcripts were then compared with the Nr database (non-redundant protein database) and nucleotide sequences in NCBI in order to identify their putative functions (Table S1). A total of 37,355 (74.94%) unigenes were annotated in the Nr database. The other unannotated transcripts represent novel genes whose functions need further investigation. According to the sequence alignment analysis, approximately 4083 (10.95%) unigenes showed the highest similarities with *Zootermophsis nevadensis* in the Nr database, followed by *Daphnia pulex* (3,166, 8.49%), *M. nipponense* (1969, 5.28%) and *Stegodyphus mimosarun* (1403, 3.76%). The unigenes were further matched with the known genomes of crustacean species, including *Litopenaeus vannamei*, *Eriocheir sinensis*, *Portunus trituberculatus*, and *Drosophila melanogaster.* A total of 3067, 3263, 3198 and 167 unigenes matched the known proteins with the genome of *L. vannamei*, *E. sinensis*, *P. trituberculatus*, and drosophila fly, respectively. Generally speaking, the unigenes of *M. nipponense* transcriptome showed the highest sequence identities with that of *E. sinensis*.

**Figure 2:**
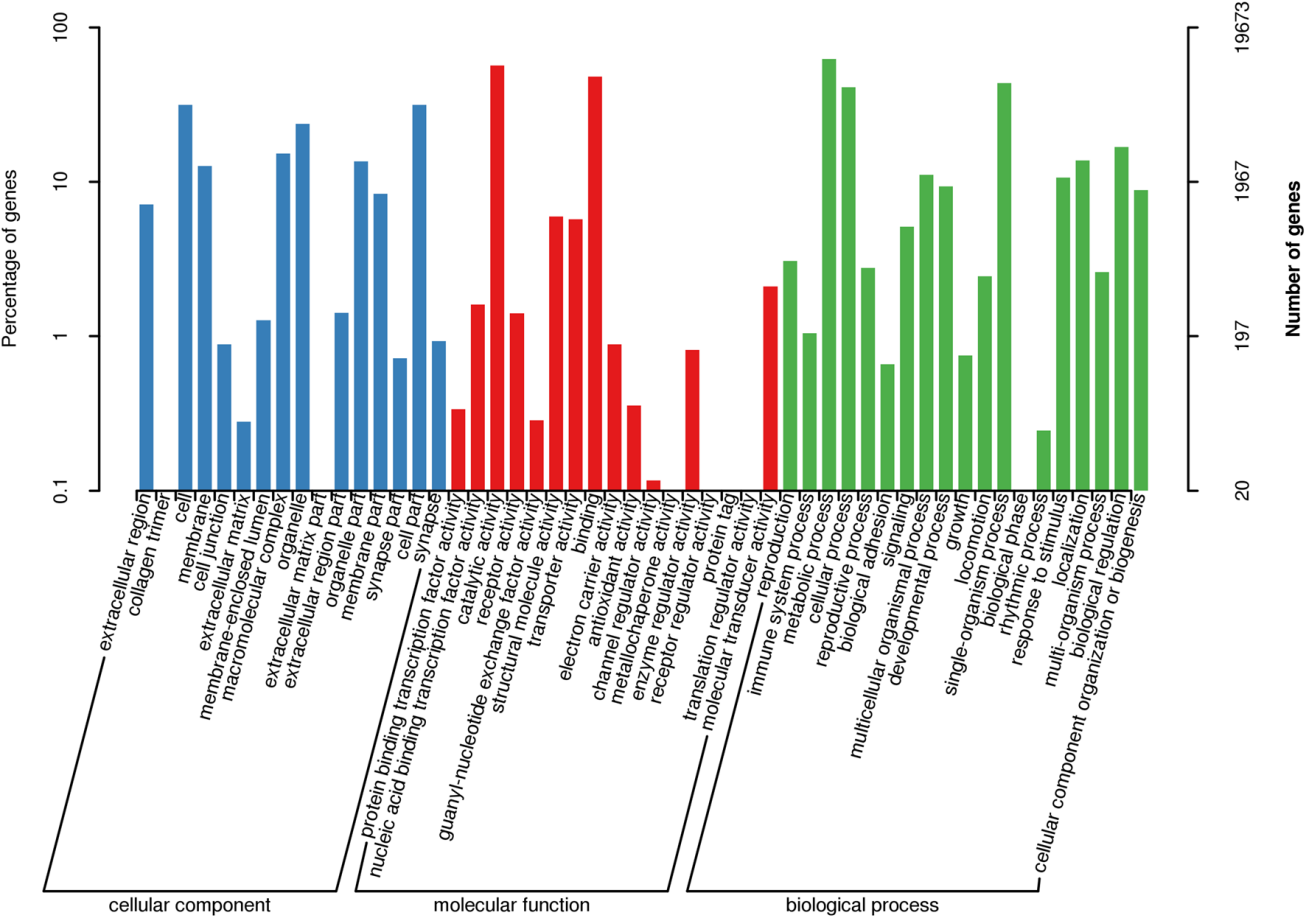
Gene ontology classification of non-redundant transcripts. Three categories of GO analysis include biological process (19 functional groups), cellular component (16 functional groups), and molecular function (17 functional groups). The left y-axis and right y-axis indicate the percentage and the number of a specific category of genes existed in the main category, respectively.

**Figure 3:**
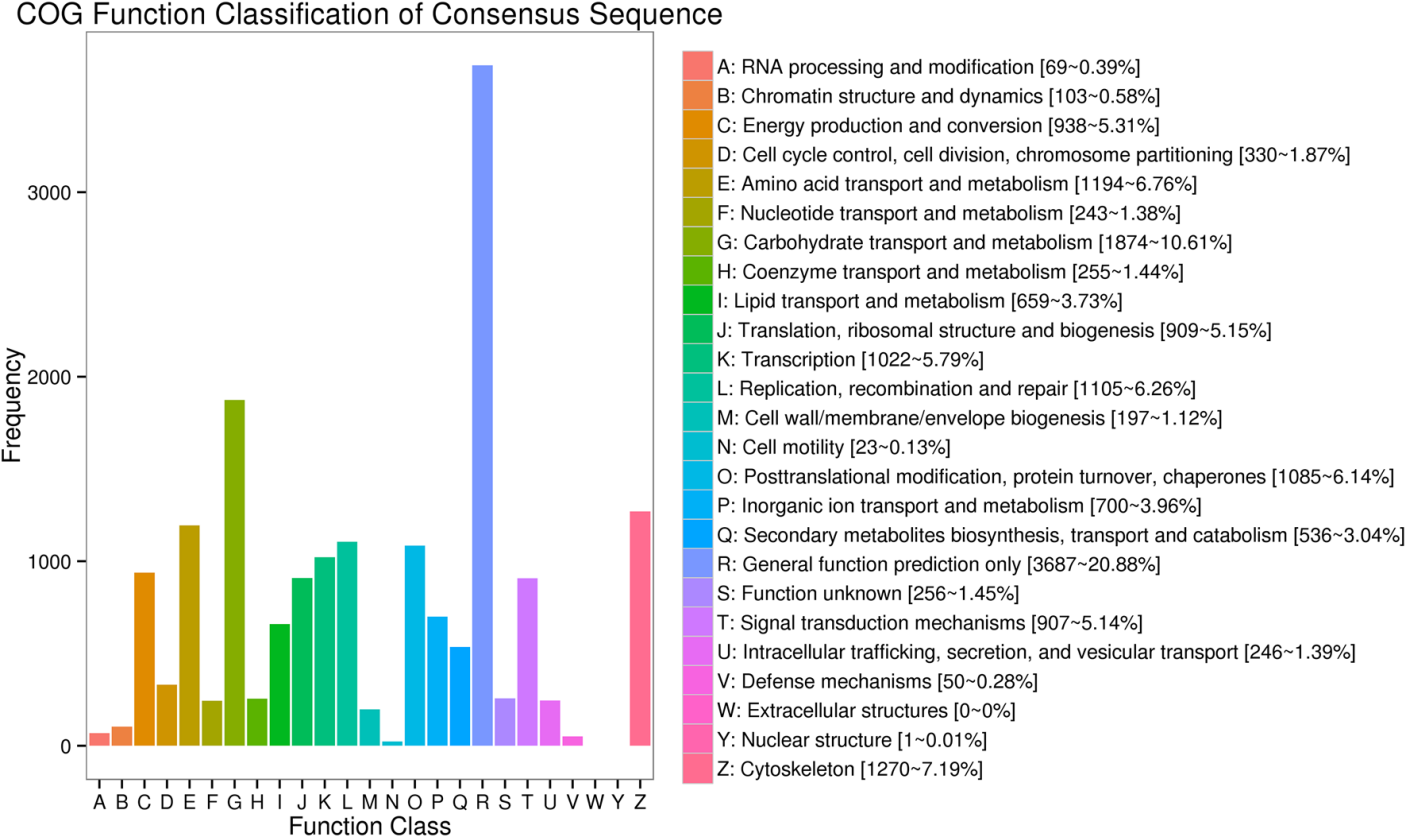
Cluster of orthologous groups (COG) classification of putative proteins.

Gene Ontology (GO) and Cluster of Orthologous Groups (COG)analysis aimed to provide a structured vocabulary to describe gene products. A total of 19,673 (39.76%) unigenes were assigned to the GO database comprised of 52 functional groups (Fig. 2). The number of unigenes in each functional group ranged from 1 to 10,057. A total of 13,395 (27.07%) unigenes were highly matched with known proteins in the COG database that were classified into 25 functional groups (Fig. 3). The number of unigenes in each functional group ranged from 1 to 6793. Kyoto Encyclopedia of Genes and Genomes (KEGG) analysis aimed to reveal the regulatory relationship between unigenes in the long-read transcriptome (www.kegg.jp/kegg/kegg1.html). A total of 18,618 (36.72%) unigenes were highly matched known genes in the KEGG database, mapped onto 264 metabolic pathways.

### Identification of differentially expressed genes

Differentially expressed genes (DEGs) were identified, using the criterion of > 2.0 as up-regulatory genes and < 0.5 as down-regulatory genes, and with a *P* value < 0.05. A total of 1319 DEGs were identified between CG and SS, including 713 up-regulated genes and 606 down-regulated genes. A total of 2092 DEGs were identified between SS and DS, including 1036 up-regulated genes and 1056 down-regulated genes. A total of 4351 DEGs were found between CG and DS, including 2163 up-regulatory genes and 2188 down-regulatory genes. KEGG analysis revealed that Cell cycle, Cellular Senescence, Oxidative Phosphorylation, Glycolysis/Gluconeogenesis and Steroid Hormone Biosynthesis were the main enriched metabolic pathways in all of these three comparisons.

A total of 15 DEGs were selected from these enriched metabolic pathways, which are listed in Table 1. These genes were differentially expressed in at least two of the three comparisons. Cyclin B3, *MAD2A*, Polo-like kinase 1, Cyclin A, cyclin-dependent kinase 2 (*Cdk2*) and Cyclin B were found in the metabolic pathways of Cell cycle and Cellular senescence, which were differentially expressed in all three comparisons. Succinate dehydrogenase complex iron sulfur subunit B Gene (*SDHB*), Cytochrome c oxidase assembly protein COX11 and Cytochrome c oxidase subunit 7A1 were selected from the metabolic pathways of Oxidative Phosphorylation. Acetyl-coenzyme A synthetase 2-like, Fructose-bisphosphate aldolase and Alcohol dehydrogenase class-3 were differentially expressed in the metabolic pathways of Glycolysis/Gluconeogenesis. Estrogen Sulfotransferase, 3 beta-hydroxysteroid dehydrogenase and *HSDL1* were identified from the metabolic pathways of Steroid Hormone Biosynthesis.

**Table 1:** Identification of important DEGs from transcriptome profiling analysis.

Name	Accession number	*P* value	CG versus SS	CG versus DS	SS versus DS	Metabolic pathways
				Fold change		
SDHB	AIC55101.1	3.07E−08	0.48	0.43		Oxidative phosphorylation; Citrate cycle
cytochrome c oxidase assembly protein COX11	XP_004522467.1	0.029	2.18	2.98		Oxidative phosphorylation; Thermogenesis
cytochrome c oxidase subunit 7A1	XP_023170779.1	1.22E−16		2.73	2.68	Oxidative phosphorylation; Thermogenesis; Parkinson disease
Acetyl-coenzyme A synthetase 2-like	XP_018428753.1	3.72E−08		2.93	2.44	Glycolysis/Gluconeogenesis; Pyruvate metabolism; Glyoxylate and dicarboxylate metabolism
Fructose-bisphosphate aldolase	XP_018019177.1	1.41E−18		2.78	2.24	Glycolysis/Gluconeogenesis; Glycerolipid metabolism;
alcohol dehydrogenase class -3	ASW35082.1	4.40E−29		3.12	2.75	Glycolysis/Gluconeogenesis; Tyrosine metabolism; Chemical carcinogenesis
estrogen sulfotransferase	AJC52502.1	5.38E−07		4.43	3.09	Steroid hormone
3 beta-hydroxysteroid dehydrogenase	XP_008216462.1	0.001		3.07	3.12	Steroid hormone; Cortisol synthesis and secretion; Aldosterone synthesis and secretion
HSDL1	ADB44902.1	1.27E−48	2.71	2.91		Steroid hormone
cyclin-B3	XP_018006504.1	1.61E−07	0.48	0.19	0.39	Cell cycle; FoxO signaling pathway; Cellular senescence
MAD2A-like	XP_023320668.1	1.09E−13	0.45	0.17	0.37	Cell cycle; Progesterone-mediated oocyte maturation; Oocyte meiosis
polo-like kinase 1	AMO03195.1	5.47E−18	0.33	0.08	0.24	Cell cycle; FoxO signaling pathway; Progesterone-mediated oocyte maturation; Oocyte meiosis
cyclin A	AGG40744.1	1.21E−15	0.49	0.15	0.31	Cell cycle; Human papillomavirus infection; Epstein-Barr virus infection; Progesterone-mediated oocyte maturation; Cellular senescence
Cdc2 kinase	ADB44904.1	1.87E−27	0.45	0.13	0.29	Cell cycle; Gap junction; Oocyte meiosis; p53 signaling pathway; Cellular senescence
cyclin B	ADB44902.1	8.92E−32	0.37	0.10	0.26	Cell cycle; Progesterone-mediated oocyte maturation; Oocyte meiosis; FoxO signaling pathway; Cellular senescence; p53 signaling pathway

### qPCR verification

**Figure 4:**
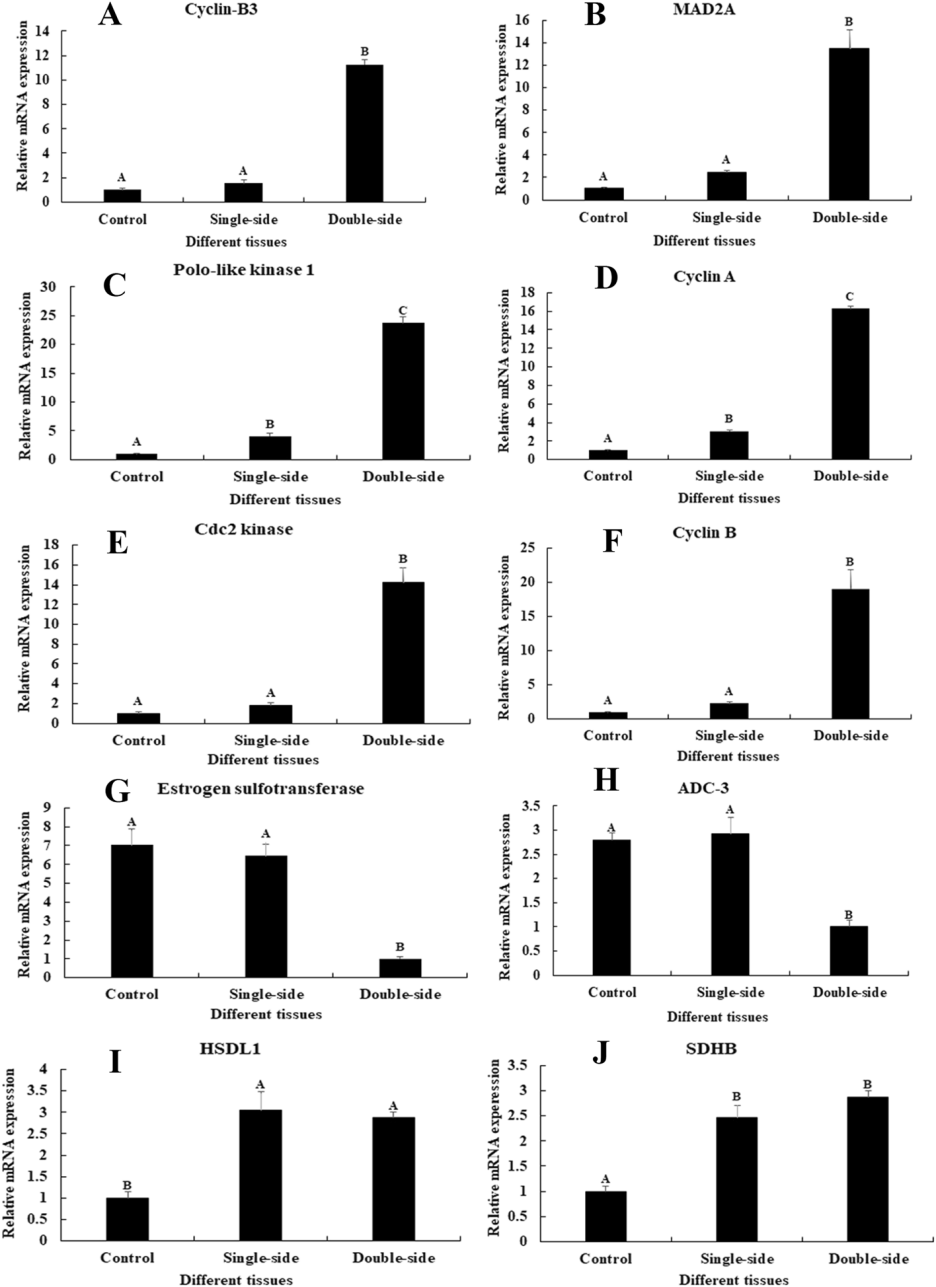
Verification of the expression of 10 differentially expressed genes (DEGs) between the androgenic gland of CG, SS and DS by qPCR. The amounts of DEGs expression were normalized to the *EIF* transcript level. Data are shown as mean ± SD (standard deviation) of tissues in three separate individuals. Capital letter indicates expression (*P* < 0.05).

qPCR analysis was used to verify the expressions of important DEGs in the androgenic gland from the CG, SS, and DS prawns. We selected 10 out of 15 DEGs to verify the accuracy of RNA-seq. The qPCR analysis showed the same expression pattern as the RNA-seq (Fig. 4). Six DEGs from the metabolic pathways of Cell Cycle and Cellular Senescence showed the lowest expressions in CG prawns, and highest expressions in DS prawns, including Cyclin B3 (Fig. 4A), *MAD2A* (Fig. 4B), Polo-like kinase 1 (Fig. 4C), Cyclin A (Fig. 4D), Cdc2 kinase (Fig. 4E) and Cyclin B (Fig. 4F). The mRNA expression of these DEGs in DS prawns showed significant difference with CG prawns and SS prawns (*P* < 0.01). The mRNA expression of estrogen sulfotransferase (Fig. 4G) and alcohol dehydrogenase class-3 (*ADC-3*) (Fig. 4H) showed no significant difference between CG prawns and SS prawns (*P* > 0.05), whereas a significant difference was observed in DS prawns (*P* < 0.05). The mRNA expression of *HSDL1* (Fig. 4I) and *SDHB* (Fig. 4J) between SS and DS prawns showed no significant difference (*P* > 0.05), whereas a significant difference was found in CG prawns (*P* < 0.05).

### Expression analysis of Mn-HSDL1

**Figure 5:**
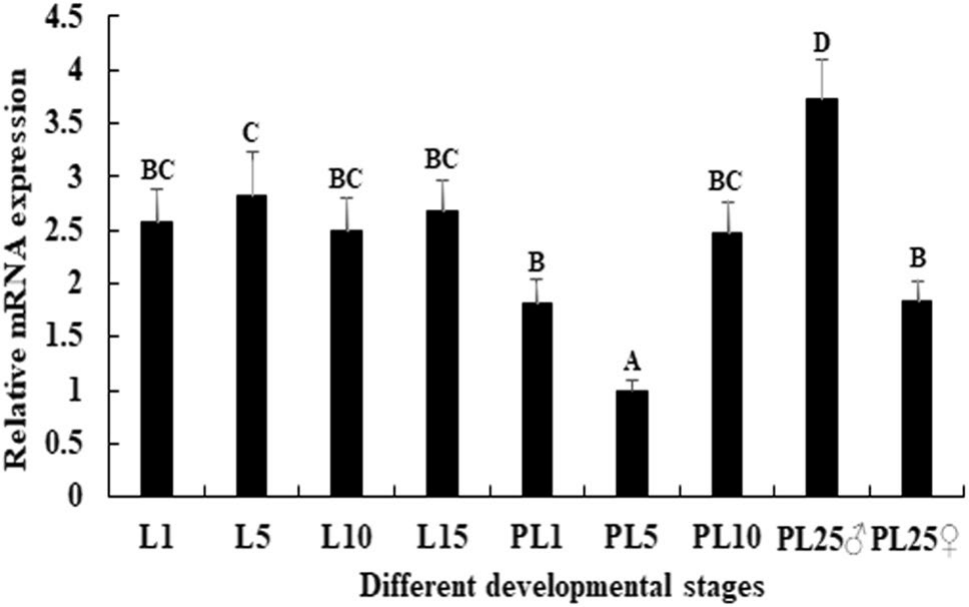
Expression characterization of *Mn-HSDL1* in different developmental stages. The amount of *Mn-HSDL1* mRNA was normalized to the *EIF* transcript level. Data are shown as mean ± SD (standard deviation) of tissues from three separate individuals*.* Capital letters indicate expression difference between different samples (*P* < 0.05).

Previous studies have shown that *Mn-HSDL1* mRNA showed the highest expression level in the hepatopancreas, followed by the testis, which showed significant difference with other tested tissues (*P* < 0.05)^38^. The mRNA expression of *Mn-HSDL1* in different prawn developmental stages was measured by using qPCR (Fig. 5). *Mn-HSDL1* expression in the larval developmental stages was generally higher than in post-larval-developmental. The highest expression level was observed in larval day 5 (L5), while it showed no significant difference with other tested stages (*P* > 0.05). During the post-larval developmental stage, the lowest expression level was observed in post-larval day 5 (PL5), and then gradually increased. The highest expression level was observed in PL25♂, which was 3.72 and 1.94-folder higher than PL5 and PL25♀, respectively.

### RNAi analysis of Mn-HSDL1

**Figure 6:**
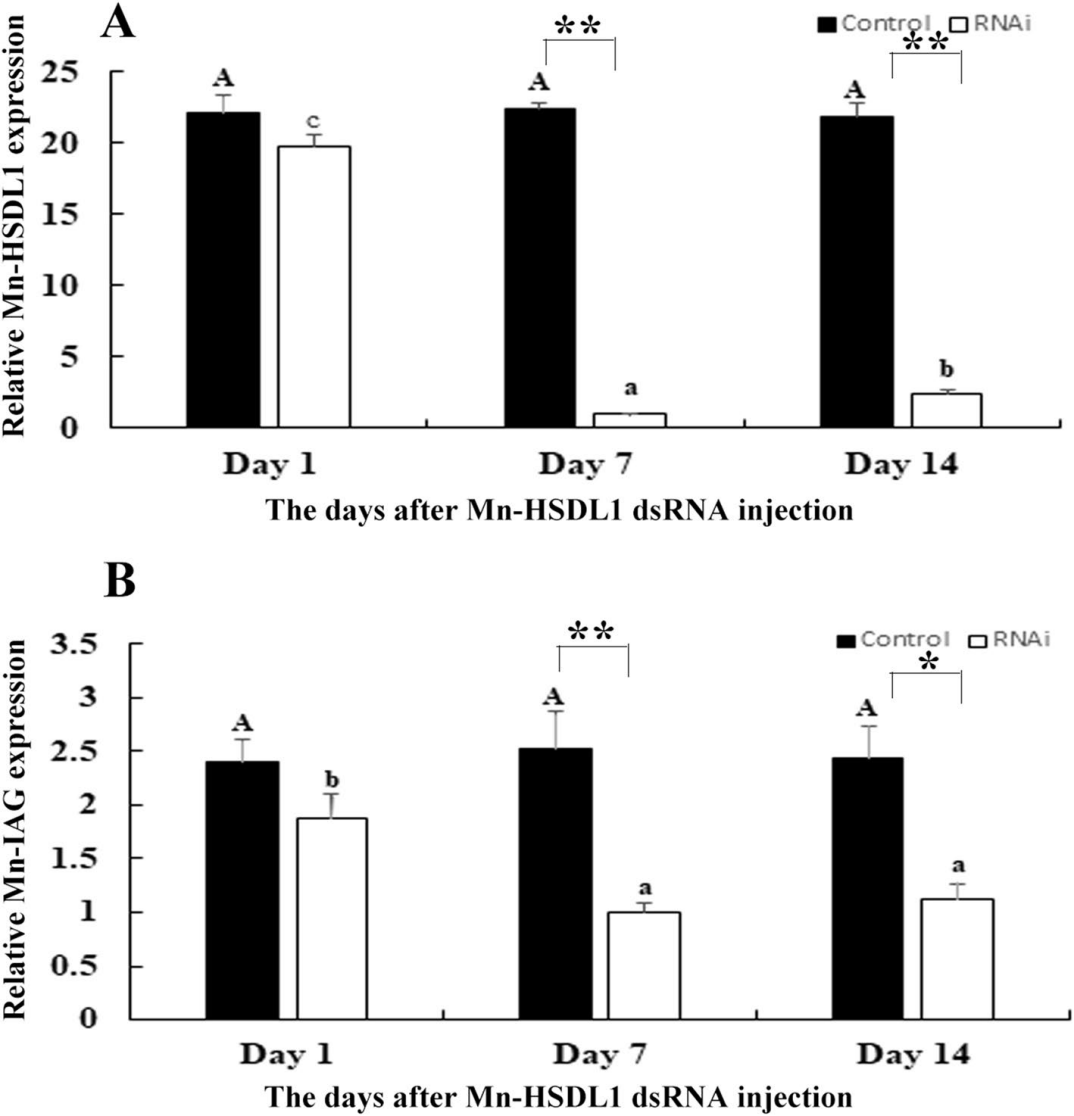
Measurement of the expression levels of *Mn-HSDL1* and *Mn-IAG* at different days after *Mn-HSDL1* dsRNA injection. EIF transcript level was used to normalize the amount of *Mn-HSDL1* and *Mn-IAG* mRNA expression. Three separate individuals were performed for each tissue, and data are shown as mean ± SD (standard deviation)*.* Capital letters and lowercase indicated expression difference between different days after GFP injection in control group and *Mn-HSDL1* dsRNA injection in RNAi group, respectively. * (*P* < 0.05) and ** (*P* < 0.01) indicates significant expression difference between the RNAi group and control group at the sample day. (**A**) Measurement of the expression levels of *Mn-HSDL1* at different days after *Mn-HSDL1* dsRNA injection. (**B**) Measurement of the expression levels of *Mn-IAG* at different days after *Mn-HSDL1* dsRNA injection.

RNAi was used to analyze the functions of *Mn-HSDL1* on male sexual development in *M*. *nipponense*. qPCR analysis revealed that the expression of *Mn-HSDL1* remained stable in the control group after the injection of *Mn-HSDL1* dsRNA (*P* > 0.05). However, the expression of *Mn-HSDL1* significantly decreased by 96% and 90% at day 7 and 14, respectively, following the injection of *Mn-HSDL1* dsRNA as compared with the control group (Fig. 6A).

The expression of *Mn-IAG* was also measured in a cDNA template of androgenic gland from the same prawns (Fig. 6B). According to the qPCR analysis, the expression of *Mn-IAG* at day 1 in the control group was slightly higher than on day 7 or day 14, when it generally remained stable (*P* > 0.05). In the RNAi group, the expression of *Mn-IAG* was significantly decreased at day 7 and day 14 after the injection of *Mn-HSDL1* dsRNA. Specifically, the expression decreased by ~ 61% and 54% at day 7 and 14, respectively, compared with the control group (*P* < 0.05).

### Histological observations of testes after RNAi

**Figure 7:**
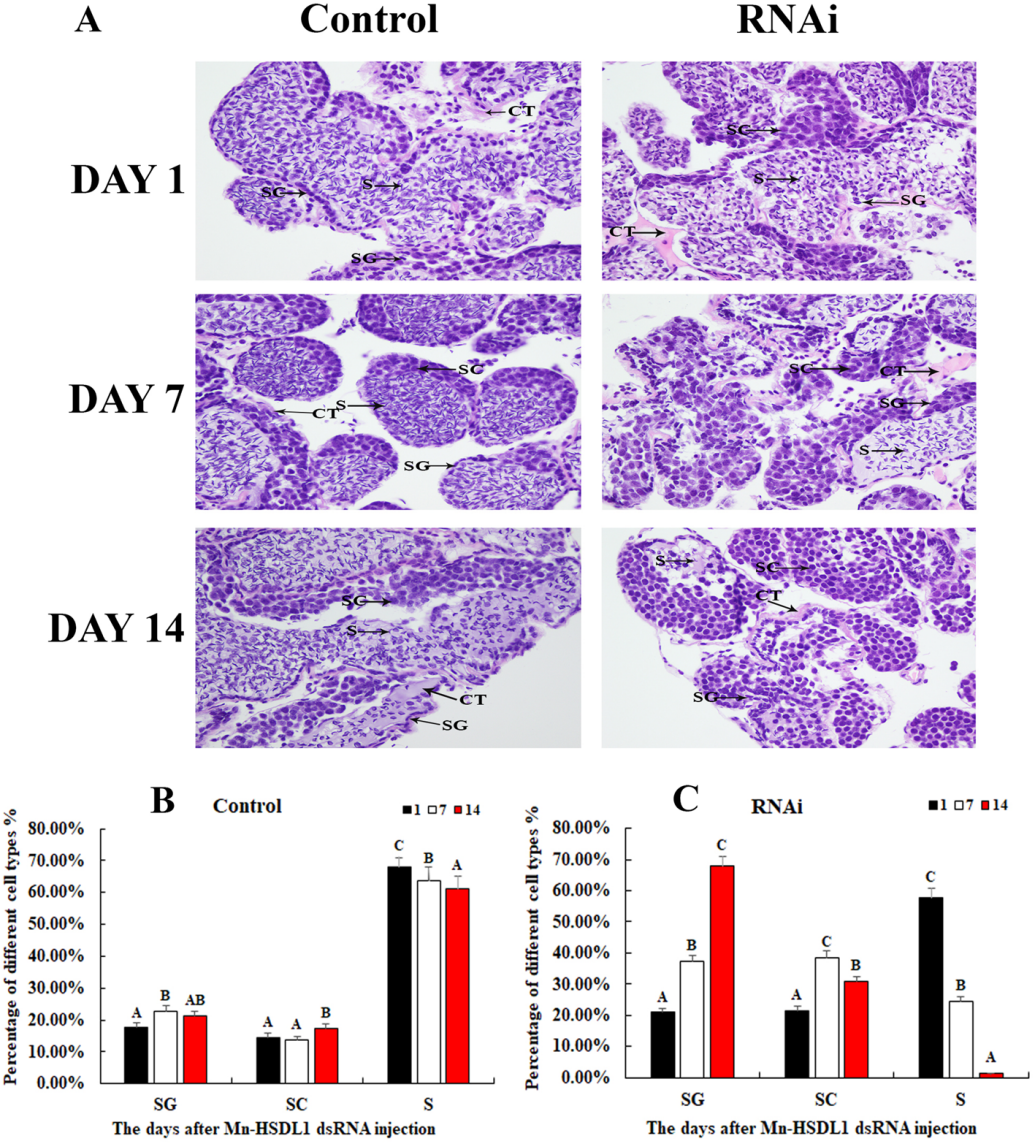
The histological observations of testis between RNAi and control group. SG: Spermatogonia; SC: spermatocyte; S: sperm; CT: collecting tissue. Scale bars = 20 μm. Data are shown as mean ± SD (standard deviation) from three separate slides*.* Capital letters indicated statistically significant percentage difference for the same cell type between different days in control group and RNAi group. (**A**) The histological observations of testis between RNAi and control group. (**B**) The percentages of different cell types in control group. (**C**) The percentages of different cell types in RNAi group.

According to histological observations, sperm was the dominant cell type in the testes from the control group, and only a limited number of spermatogonia and spermatocytes were observed (Fig. 7A). The percentages of sperm in Day 1, 7 and14 of control group were 67.90%, 63.64% and 61.24%, respectively (Fig. 7B). In the RNAi group, the number of sperm gradually deceased with the time of *Mn-HSDL1* dsRNA treatment. Sperm were rarely found at day 14 after *Mn-HSDL1* dsRNA treatment. The percentages of sperm decreased from 57.69% at Day 1 to 1.27% at Day 14 in RNAi group (Fig. 7C). However, the number of spermatogonia increased from 20.85% at Day 1 to 67.89% at Day 14 in RNAi group (Fig. 7C).

### Regulatory effects of *Mn-HSDL1* with *IGF1, IGF2, CYP11* and *PRKAA2*

**Figure 8:**
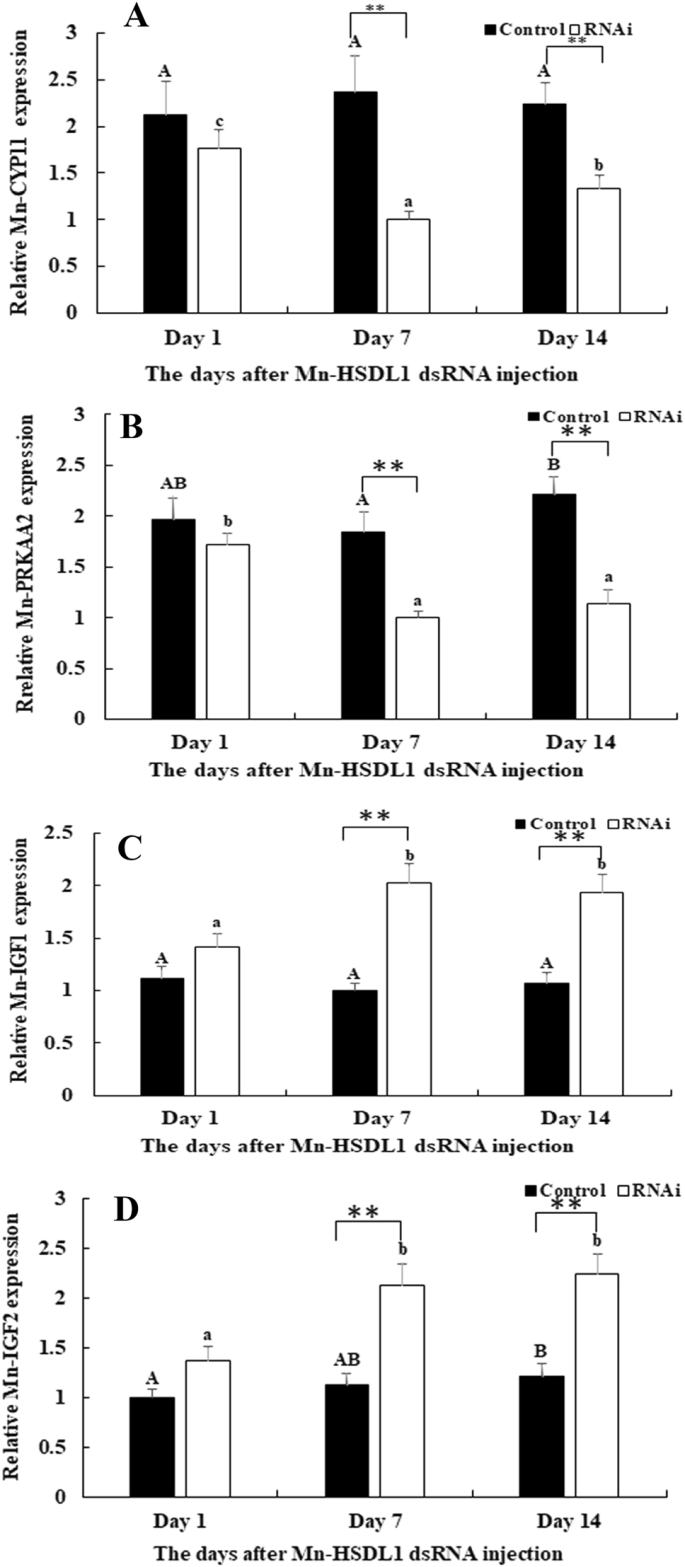
Expression characterization of *Mn-CYP11*, *Mn-PRKAA2*, *Mn-IGF1* and *Mn-IGF2* at different days after *Mn-HSDL1* dsRNA injection. The amount of mRNA expression was normalized to the *EIF* transcript level. Data are shown as mean ± SD (standard deviation) of tissues from three separate individuals*.* Capital letters indicate statistically significant expression differences between different days after GFP dsRNA injection in control group. Lowercase indicated expression difference between different days after *Mn-HSDL1* dsRNA injection in the RNAi group. ** (*P* < 0.01) indicates a significant expression difference between the RNAi group and control group at the sample day. (**A**) Expression characterization of *Mn-CYP11*. (**B**) Expression characterization of *Mn-PRKAA2*. (**C**) Expression characterization of *Mn-IGF1*. (**D**) Expression characterization of *Mn-IGF2*.

*HSDL1* was reported to have regulatory relationship with that of Insulin-like growth factor 1 (*IGF1*), Insulin-like growth factor 2 (*IGF2*), Cytochrome P450 (*CYP11*) and 5′-AMP-activated protein kinase catalytic subunit alpha-2 (*PRKAA2*) in the previous studies^39,40^. The regulatory effects of *Mn-HSDL1* with *Mn-IGF1*, *Mn-IGF2*, *Mn-CYP11* and *Mn-PRKAA2* were measured in the same cDNA template of RNAi by using qPCR. According to the qPCR analysis, the expressions of *Mn-CYP11* and *Mn-PRKAA2* were decreased with the decrease of *Mn-HSDL1*, which showed positive regulatory effects (Fig. 8A,B). However, the expressions of *Mn-IGF1* and *Mn-IGF2* were increased with the decrease of *Mn-HSDL1*, which showed negative regulatory effects (Fig. 8C,D).

## Discussion

The eyestalk of crustaceans secretes many neurosecretory hormones that mediate reproduction, molting and metabolism of glucose in crustaceans^23–34^. The important neurosecretory hormones include *CHH*, *ITP*, *GIH*, *MIH* and *MOIH*. This study aimed to analyze the effects of eyestalk hormones on male sexual development. qPCR analysis revealed that the mRNA expression of *Mn-IAG* significantly increased at day 4 and day 7 after eyestalk ablation in both SS group and DS group, compared with day 1 (Fig. 1). The expression in DS group was significantly higher than SS group, which is consistent with previous studies^41–43^. However, the expression between day 4 and day 7 showed no significant difference in both SS group and DS group. *IAG* has been reported to promote male sexual differentiation and development in crustaceans^8–10^. Thus, the increase in *Mn-IAG* expression after the eyestalk ablation indicated that the eyestalk has negative effects on male sexual differentiation and development in *M*. *nipponense*. This also has a similar mediated function in ovarian development in *M*. *nipponense*^35^.

To date, this is the first long-read transcriptome in *M*. *nipponense*. The combination of long-read and next generation transcriptome sequencing can be useful to obtain transcripts with better integrity and quality for further gene structure and functional analysis. The accuracy and length of the transcripts of long-read transcriptome are further improved and optimized through correction by the next generation transcripts. Thus, this method is a suitable strategy for a species without a reference genome. The genes related to male sexual development were predicted to be found in the functional groups of Cell, Cellular Process and Binding in the GO assignment (Fig. 2), and the functional groups of General Function prediction only, Signal Transduction Mechanisms, Posttranslational Modification, Protein Turnover and Chaperones in the COG classification (Fig. 3), which were consistent with previous studies^38,44^. The gene sequences from this long-read transcriptome provide valuable information for the analysis of gene structure and function.

The number of DEGs between the CG versus DS groups were 4351, which were significantly greater than the number of DEGs between CG versus SS and SS versus DS. This indicates that the ablation of the double-side eyestalk has more regulatory roles on male sexual development in *M*. *nipponense*. This was consistent with the qPCR analysis. KEGG analysis revealed that Cell cycle, Cellular Senescence, Oxidative Phosphorylation, Glycolysis/Gluconeogenesis and Steroid Hormone Biosynthesis were the primary enriched metabolic pathways in all three comparisons. Previous studies have predicted the important roles of Oxidative Phosphorylation, Glycolysis/Gluconeogenesis and Steroid Hormone Biosynthesis in the mechanism of male sexual development in *M*. *nipponense*^38,44^. These previous studies included the transcriptome profiling analysis of testis between reproductive season and non-reproductive season^38^, and transcriptome analysis during the sex-differentiation and development sensitive period^44^. In these reports, it was predicted that the DEGs from these particular metabolic pathways play function in male sexual development in *M*. *nipponense* by providing ATP and promoting the biosynthesis of steroid hormones. The present study revealed that several metabolic pathways and DEGs involved in the immune system are predicted to participate in the mechanism of male sexual development in *M. nipponense*. These included Cell Cycle and Cellular Senescence. A reasonable explanation is that vigorous male sexual development after ablation of the eyestalks is due to a significant increase in *IAG* expression. Thus, immune related metabolic pathways are needed, in order to maintain normal testes development. It is suggested that these metabolic pathways digest aged cells and prevent proliferation of damaged copies of DNA.

The transcriptome profiling analysis revealed that Cell Cycle and Cell Senescence are the most enriched metabolic pathways in all three comparisons. The Cell Cycle is a ubiquitous and complex process that ensures correct cell proliferation. This pathway is crucial for the prevention and/or correction of damaged DNA, genetic abnormalities and mutations, with cyclins and cyclin-dependent kinases functioning in this process^45,46^. Cellular Senescence is defined as irreversible cell cycle arrest caused by different forms of stress. These stresses include telomere shortening, genotoxic stress, mitogens or inflammatory cytokines, the activation of the p53 tumor suppressor gene and/or the cyclin-dependent kinase inhibitor p16^47,48^. The dramatic enrichment of DEGs in these two metabolic pathways indicates that Cell Cycle and Cell Senescence function in the proofreading process when cells undergo replication. Four DEGs were enriched in both of the Cell Cycle and Cell Senescence categories, including cyclin A, cyclin B, cyclinB3 and *Cdk2*. Cyclin A is a vital component of the cell-cycle machinery, which can activate two different cyclin-dependent kinases (*Cdk1* and *Cdk2*), functioning in both S-phase and mitosis^49–51^. Cdk1/cyclin B, also known as maturation promoting factor (*MPF*), is one of the main protein kinases. It activates, and serves as master regulator, for the M-phase transition, phosphorylating and activating other downstream protein kinases, and directly phosphorylating several structural proteins involved in cellular reorganization^52–54^. The *Cdk* family includes eight *Cdk* genes that can combine with different types of cyclins to form complexes, regulating the process of cell transition from the G1 phase to the S phase or G2 phase to the M phase and finally exiting from M phase. *Cdk2* in particular is a member of a highly conserved family of protein kinases, regulating the eukaryotic cell cycle^55–57^.

Adenosine-triphosphate (ATP), a high-energy compound used as an energy source in nearly all metabolic activities, is essential for male differentiation and development. Therefore, it is of interest that in the present study, Oxidative Phosphorylation and Glycolysis/Gluconeogenesis were the main enriched metabolic pathways in all three comparisons. Oxidative Phosphorylation occurs in the inner membrane of mitochondria of eukaryotic cells or in the cytoplasm of prokaryotes. The energy released from the oxidation of substances in vivo promotes the coupling reaction between adenosine diphosphate (ADP) and inorganic phosphate to synthesize ATP through the respiratory chain^58^. Glycolysis/Gluconeogenesis promotes the conversion of glucose (C_6_H_12_O_6_) into pyruvate (CH_3_COCOO−  + H+), releasing free energy to form ATP and reduced nicotinamide adenine dinucleotide^59^. Three DEGs were selected from Oxidative Phosphorylation and Glycolysis/Gluconeogenesis. SDHB, a DEG that was down-regulated between CG versus SS and CG versus DS. *SDHB*, was also predicted to be involved in the mechanism of male sexual development in *M*. *nipponense*^38^. *SDHB* is one of four protein subunits that form succinate dehydrogenase, which catalyzes the oxidation of succinate^60,61^. Two subunits of cytochrome c oxidase, which function during oxidative phosphorylation, were also differentially expressed. These two subunits included cytochrome c oxidase assembly protein COX11 and cytochrome c oxidase subunit 7A1. Cytochrome c oxidase is located at the end of the cytochrome c system in cellular respiration. This enzyme directly transfers the electrons of respiratory substrates to molecular oxygen through the cytochrome system^62,63^.

It is widely acknowledged that steroid hormones primarily function in sexual development^64,65^. Hormones are generally divided into five main classes: glucocorticoids, mineralocorticoids, androgens, estrogens, and progestogens. Natural steroid hormones, which are lipids, are generally synthesized from cholesterol in the gonads and adrenal glands^66,67^. *HSDL1* was differentially expressed between CG versus SS and CG versus DS, indicating that the expressions of *HSDL1* is significantly regulated by the ablation of both single-side eyestalk and double-side eyestalk. *HSDL1* was also shown to be involved in the mechanism of male sexual development in a previous study^38^. The short-chain dehydrogenase/reductases family (SDR) is a large enzyme family, which can affect mammalian reproduction, hypertension, neoplasia, and digestion^68,69^. Hydroxysteroid dehydrogenase is a subfamily of SDR, that functions in sex-determination, establishment and maintenance of secondary sexual characteristics, and the regulation of the endocrine system through catalyzing the metabolism of steroid hormones. *HSDL1* is an important gene in the metabolic pathway of steroid hormones^70^. qPCR verification revealed that the expression pattern of important DEGs from these metabolic pathways were the same as the RNA-seq data (Fig. 4). This is an indicator of the accuracy of the transcriptome profiling analysis.

Both this study and previous studies predict the potentially vital roles of *HSDL1* in the mechanism of male sexual development in *M*. *nipponense*^38^. Thus, the function of *HSDL1* in male sexual development was also analyzed by using qPCR and RNAi, combined with histological observations. Previous studies have shown that *HSDL1* was highly expressed in reproductive tissues (i.e., testes and ovaries) in humans, as revealed by Northern Blot analysis^70^. In situ hybridization indicated that the expression of *HSDL1* was higher in prostate cancer than that in normal prostate tissue. In addition, this gene is involved in the fetal sheep development in the late gestational stages^71^. The qPCR analysis in different mature tissues revealed that the highest expression level of *Mn-HSDL1* was observed in hepatopancreas, followed by testes, while *Mn-HSDL1* RNA was rarely detected in other tissues^38^. Thus, it is predicted that HSDL1 may be involved in testes development in *M. nipponense*. RNAi was further used to analyze the potential functions of *Mn-HSDL1* in testes development. The mRNA expression of *Mn-HSDL1* was significantly decreased at day 7 and day 14 after *Mn-HSDL1* dsRNA injection (Fig. 6A), indicating the RNAi used was effective in this study. The mRNA expression of *Mn-IAG* was also measured in the androgenic gland from the same prawn (Fig. 6B). The qPCR analysis revealed that *Mn-IAG* expression decreased with the decrease of *Mn-HSDL1*, indicating that *HSDL1* has a positive regulatory effect on *IAG* in *M*. *nipponense*. *IAG* is a hormone, secreted by androgenic gland, promoting male sexual differentiation and development in many crustacean species^8–10^. According to the histological observations, the number of sperm decreased with the time of *Mn-HSDL1* dsRNA injection. Compared with the control group, sperm were rarely found at day 14 after *Mn-HSDL1* dsRNA injection (Fig. 7). This indicated that *HSDL1* has a positive regulatory effect on testes development in *M*. *nipponense*. In different developmental stages, expression in the larval developmental stages was generally higher than in the post-larval developmental stages, indicating that *HSDL1* may be involved in organ development and metamorphosis of *M*. *nipponense*. This is particularly observed in the development of the hepatopancreas, heart, and gills^72,73^, however, this requires further investigation. *Mn-HSDL1* mRNA expression gradually increased from PL5 to PL25. The period from PL5 to PL25 is the sex-differentiation sensitive period^5^. Thus, the increase from PL5 to PL25 indicates that *HSDL1* might function in gonad differentiation and development. In addition, gender is first distinguished at PL25. Expression in PL25♂ was two-fold higher than PL25♀, which also indicates that *HSDL1* has a functional role in male sexual development.

Previous studies reported that HSDL1 has a regulatory relationship with *IGF1*, *IGF2*, *CYP11* and *PRKAA2*, which participate in the glycolytic and lipogenic pathway^39,40^. This study identified the positive regulatory effects of *Mn-HSDL1* on the expression of *Mn-CYP11* and *Mn-PRKAA2*. *CYP11a*, which has possible relation to hyperandrogenemia, was reported to be associated with both polycystic ovary syndrome and total testosterone levels in women with polycystic ovary syndrome^74^. *PRKAA2* is a gene that belongs to the AMP-activated protein kinase (*AMPK*). It received considerable attention because of the activation of both glucose and lipid metabolism and improvement of the insulin sensitivity^75,76^. However, *Mn-HSDL1* negatively affects expression of *Mn-IGF1* and *Mn-IGF2*. A reasonable explanation for this is that silencing *Mn-HSDL1* inhibited testis development. Thus, the energy for testis development was used to promote the growth performance^77,78^.

In conclusion, candidate genes that are possibly involved in the male sexual development were selected through performing long-read and next generation transcriptome sequencing of the androgenic gland after eyestalk ablation in *M*. *nipponense*. qPCR analysis revealed that *Mn-IAG* expression significantly increased after the ablation of both single-side and double-side eyestalks, indicating that the ablation of eyestalk has potential regulatory roles on the process of male sexual development in *M*. *nipponense*. The long-read transcriptome generated 49,480 non-redundant transcripts. A total of 1319, 2092 and 4351 DEGs were identified between CG versus SS, SS versus DS and CG versus DS, respectively, indicating that the ablation of double-side eyestalks plays a regulatory role on male sexual development. Cell Cycle, Cellular Senescence, Oxidative Phosphorylation, Glycolysis/Gluconeogenesis and Steroid Hormone Biosynthesis were the main enriched metabolic pathways in all three comparisons, and the important DEGs from these metabolic pathways were identified. qPCR analysis and RNAi analysis of *Mn-HSDL1* indicated that *HSDL1* has positive regulatory effects on testes development. Overall, this study provided valuable information concerning the mechanisms underlying male sexual development in *M*. *nipponense* and potentially other crustacean species as well.

## Materials and methods

### Ethics statement

Permission was obtained from the Tai Lake Fishery Management Council and the committee of Freshwater Fisheries Research Center during the experimental programs. MS222 anesthesia was used to sedate the prawns and shear the tissues. All experiments were performed in accordance with relevant guidelines and regulations. Authors complied with the ARRIVE guidelines.

### Sample collection

A total of 600 healthy male prawns and 20 healthy female prawns of *M*. *nipponense* were collected from a wild population in Tai Lake in July, Wuxi, China (120° 13′ 44″ E, 31° 28′ 22″ N). The body weight of male prawns was 3.63–4.94 g and the body weight for females was 3.21–3.45 g. All samples were randomly divided and transferred to three, 500 L tanks and maintained in aerated freshwater for three days. The three groups in this study were: CG, SS, and DS. The androgenic glands were collected from the three groups after 7 days of eyestalk ablation, and immediately preserved in liquid nitrogen until used for long-read and next-generation transcriptomic analysis. Mature tissues that were studied included testes ovaries, hepatopancreas, muscle, eyestalk, gill, heart and brain. One male parent prawn with a body weight of 4.87 g and one female parent prawn with a body weight of 3.45 g were collected from the wild population and mated in the laboratory in order to produce the full-sibs population. Specimens for the different stages of larval and post-larval developmental stages were obtained from the full-sibs population after hatching and collected throughout the maturation process.

### Long-read transcriptome analysis

In order to provide sufficient RNA with an aim to establish a reference transcriptome for further analysis, equal amount of androgenic gland tissue from the CG, SS, and DS groups (N ≥ 60) were pooled together to perform the long-read sequencing. According to the manufacturer’s instructions, the UNlQ-10 Column Trizol Total RNA Isolation Kit (Sangon, Shanghai, China) was used to extract total RNA, and an Agilent RNA 6000 Nano kit and chips on a Bioanalyzer 2100 (Agilent Technologies, Santa Clara, CA, USA) was used to measure the RNA integrity. A PacBio RSII platform (Pacific Bioscience Inc., Menlo Park, CA, USA) was employed to construct the long-read transcriptome. The detailed procedures for the construction of long-read transcriptome and the analysis of raw sequence data have been well described in our previous study^79^.

In the next step, the contaminant sequences were removed by stepwise CLC^80^, and the LRS isoforms were annotated^81^. Using Blastp, the transcriptome factors were aligned to the PlnTFDB database (http://plntfdb.bio.uni-potsdam.de/v3.0/), the AnimalTFDB database (http://bioinfo.life.hust.edu.cn/AnimalTFDB/), and the CARD database (https://card.mcmaster.ca/) for the selection of genes involved in the mechanism of male sexual development in *M*. *nipponense*, using the threshold of E-value ≥ 1e^–10^. Finally, all Blastp results were processed with BLAST2GO^82^ for functional annotation. The long-read were annotated in the M. nipponense genome by using Lorean^83^.

### Transcriptomic profiling analysis

The comparative transcriptome analysis of the androgenic gland between the CG, SS and DS groups were performed. In order to ensure the sufficient amount of RNA samples, androgenic glands from at least 30 prawns were pooled to form one biological replicate, and three biological replicates were sequenced for all three groups. Previously published studies have described the experimental process^16,42^.

Clean reads were assembled into non-redundant transcripts by using the Trinity program (version: trinityrnaseq_r20131110)^84^. The NR protein, the GO, the COG and the KEGG database were then used to perform the gene annotation, using an E-value cut-off of 10^−5^^16^. Blast2go software was used for functional annotation by GO terms^82^. Blast software was employed to perform the functional annotation against the COG^85^ and KEGG^86^ database. EB-seq algorithm was used to filter the differentially expressed genes, under the criteria of FDR (False discovery rate) < 0.05^87^.

### qPCR analysis

qPCR was used to measure the relative mRNA expression of *Mn-HSDL1* in different developmental stages, as well as for confirmation of DEGs. The Bio-Rad iCycler iQ5 Real-Time PCR System (Bio-Rad) was used to carry out the SYBR Green RT-qPCR assay. The procedure has been well described in previous studies^21,22^. The primers used for qPCR verification of important DEGs are listed in Table 2. The primers used for qPCR analysis of *Mn-HSDL1* are listed in Table 3. EIF was used as a reference gene in this study^88^. Three replicates were performed for each tissue.

**Table 2:** Primers used for qPCR verification.

Primer	Sequence
Cyclin B3-F	TGATGAAAGAACTCCGCCGT
Cyclin B3-R	AGCGCACCTGGCATATCTTC
MAD2A-F	ACCCTCCTGAGTCCTTCACTT
MAD2A-R	TGCACATGTCCTGCCTCAAG
Polo-F	CGAACTACATCGCCCCAGAA
Polo-R	AGCGGTCCAATTCTCGAAGG
Cyclin A-F	CTGCCTCATCAGTTGCGTTG
Cyclin A-R	AGCTGTGATACCGAATGCCA
Cdc2-F	ATCAGCGCAGAGTTCTTCACA
Cdc2-R	GAAGAACTTCAGGTGCACGG
Cyclin B-F	TGGGAGATGTGGGAAATCGG
Cyclin B-R	CCTCAACCTTCGCTTCTTGC
Estrogen-F	CTGCAAAACTGGCGGTCAAA
Estrogen-R	CGAGACCTGGGACGTCATTC
Alcohol-F	CCTTCCTCCAGGGACTCGTA
Alcohol-R	CCTCATACGACTGACGACCG
SDHB-F	ACCGCAAGAAGTTGGATGGT
SDHB-R	TCGATGATCCAACGGTAGGC
PDHE1-F	AGCCTAAGCGTTCCAACTCC
PDHE1-R	TATTCAGCAGACCTCGTGGC

**Table 3:** Primers used for HSDL1 analysis.

Primer name	Nucleotide Sequence (5′ → 3′)	Purpose
HSDL1-RTF	AGCCTAAGCGTTCCAACTCC	FWD primer for HSDL1 expression
HSDL1-RTR	TATTCAGCAGACCTCGTGGC	RVS primer for HSDL1 expression
IGF1- RTF	GAGGCGAAAGTCCTGTTCCA	FWD primer for IGF1 expression
IGF1- RTR	ACTCCTTAGATCGCCCCACT	RVS primer for IGF1 expression
IGF2- RTF	ATGGGCATGTACGGCTCTTC	FWD primer for IGF2 expression
IGF2- RTR	TGCAATTTTCACCGATGCCC	RVS primer for IGF2 expression
CYP11- RTF	TAACGAACCCTGACGACTGC	FWD primer for CYP11 expression
CYP11- RTR	GGGTACGGACTCTCCTCCAT	RVS primer for CYP11 expression
PRKAA2- RTF	GATTCGGGAGTTCCTAGCGG	FWD primer for PRKAA2 expression
PRKAA2- RTR	CGTCACCTCTCTCGCTTGTT	RVS primer for PRKAA2 expression
EIF-F	CATGGATGTACCTGTGGTGAAAC	FWD primer for EIF expression
EIF-R	CTGTCAGCAGAAGGTCCTCATTA	RVS primer for EIF expression
HSDL1 RNAi-F	TAATACGACTCACTATAGGGGCAGACTTCTCCAACGGAAG	FWD primer for RNAi analysis
HSDL1 RNAi-R	TAATACGACTCACTATAGGGGCAGAGCTTAACGGATGAGG	RVS primer for RNAi analysis

### RNA interference (RNAi) analysis

RNAi was performed to analyze the potential regulatory roles of *Mn- HSDL1* in male sexual development in *M*. *nipponense*. The Snap Dragon tool was used to design the specific RNAi primer with the T7 promoter site (http://www.flyrnai.org/cgibin/RNAifind_primers.pl) shown in Table 1. The Transcript Aid™ T7 High Yield Transcription kit (Fermentas, Inc, USA) was used to synthesize the *Mn-HSDL1* dsRNA, according to manufacturer’s instructions. A total of 300 healthy mature male *M*. *nipponense* with a body weight of 3.21–4.78 g were collected and divided into two groups. As described in the previous study^89,90^, prawns from the experimental group were injected with 4 μg/g *Mn- HSDL1* dsRNA, while prawns from the control group were injected with an equal volume of GFP dsRNA (control). *HSDL1* mRNA expression was investigated in the androgenic gland by qPCR 1, 7 and 14 days after the injection, permitting confirmation of silencing efficiency (N ≥ 5). mRNA expression of *Mn-IAG* was measured in the same cDNA templates in order to analyze the regulatory relationship between *Mn-HSDL1* and *Mn-IAG*.

### Histological observation

The morphological changes in the testes between different days after RNAi treatment were observed by Hematoxylin and eosin (HE) staining. Five testicular samples were collected after 1, 7, and 14 days of RNAi treatment for HE staining. The procedures have been well described in previous studies^91,92^. Olympus SZX16 microscope was used to observe the slides (Olympus Corporation, Tokyo, Japan). The various cell types were labeled based on morphological analysis^5^.

### Statistical analysis

Quantitative data were expressed as mean ± SD. Statistical differences were estimated by one-way ANOVA followed by LSD and Duncan’s multiple range test. All statistics were measured using SPSS Statistics 23.0. A probability level of 0.05 was used to indicate significance (*P* < 0.05).

## Supplementary Information

Supplementary Figure 1.

Supplementary Figure 2.

Supplementary Figure 3.

Supplementary Figure 4.

Supplementary Figure 5.

Supplementary Figure 6.

Supplementary Table S1.

## Data Availability

The reads of *M*. *nipponense* transcriptome were submitted to NCBI with the accession number of PRJNA533885.
