# Supplementary figures and images for: Identification of candidate genes from androgenic gland in Macrobrachium nipponense regulated by eyestalk ablation
Source: Sci Rep. 2021 Oct 6;11:19855. doi: 10.1038/s41598-021-99022-4 (PMC8494903; doi:10.1038/s41598-021-99022-4)

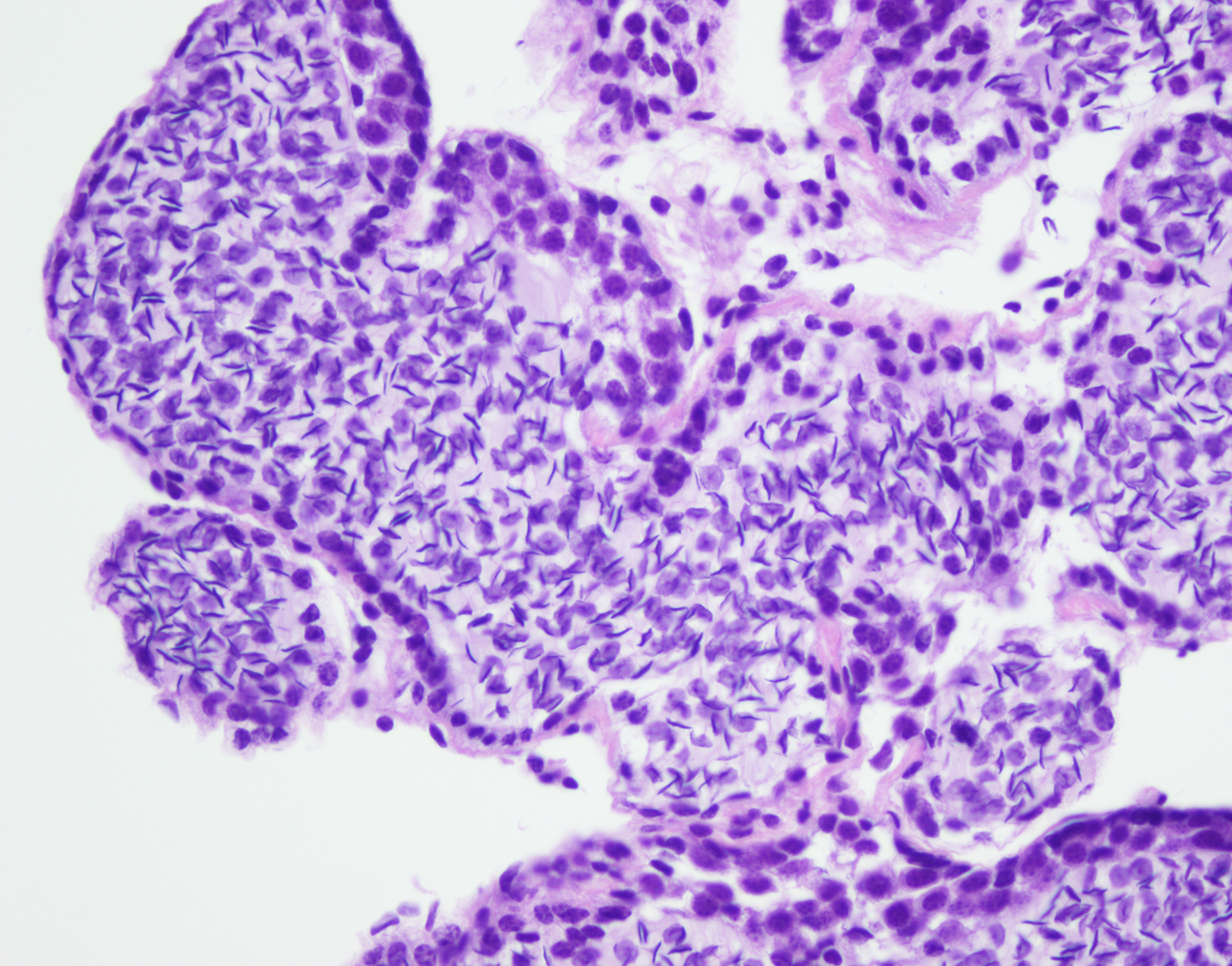

Supplement: Supplementary file 1 — Supplementary Figure 1. [file 41598_2021_99022_MOESM1_ESM.jpg]

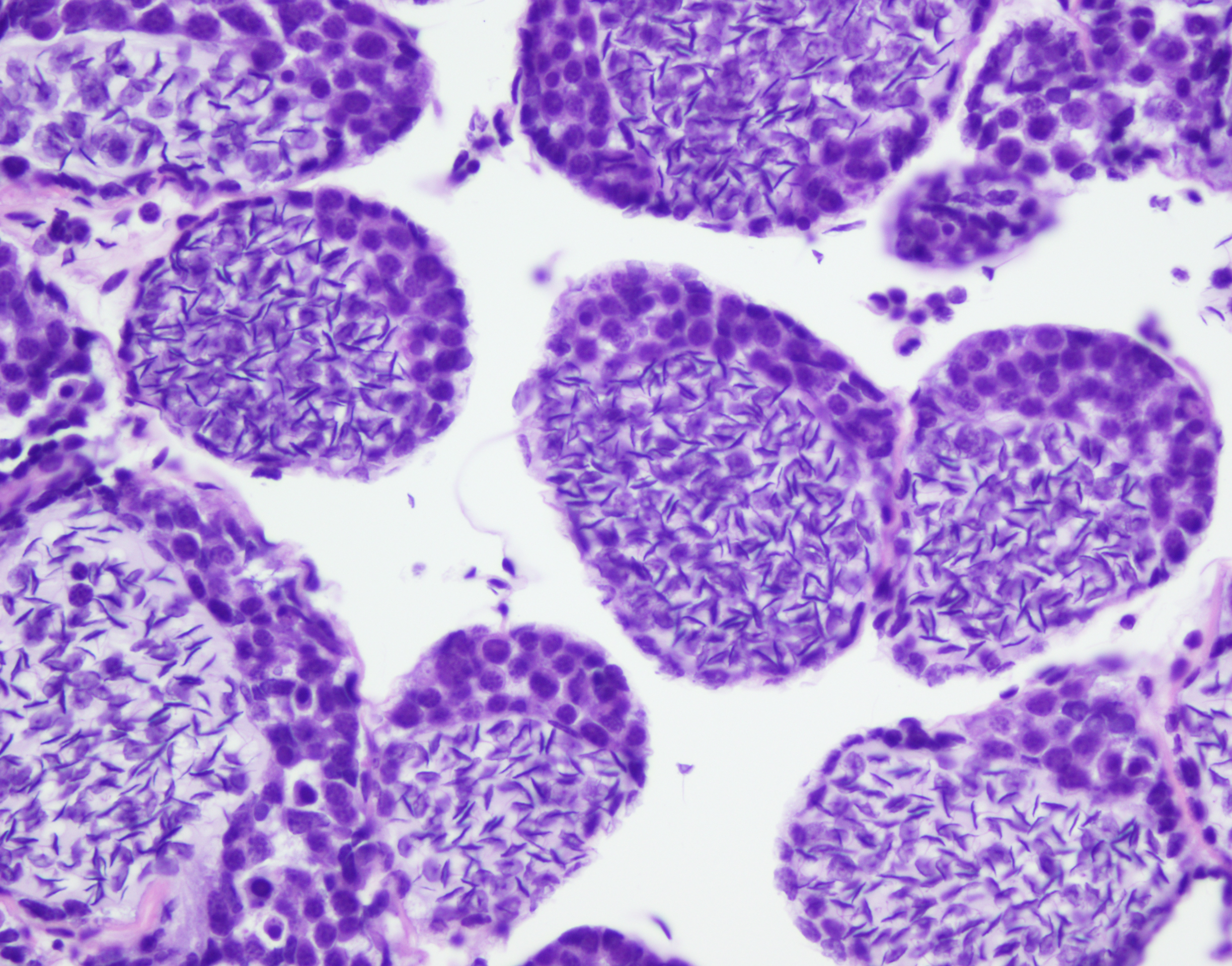

Supplement: Supplementary file 2 — Supplementary Figure 2. [file 41598_2021_99022_MOESM2_ESM.jpg]

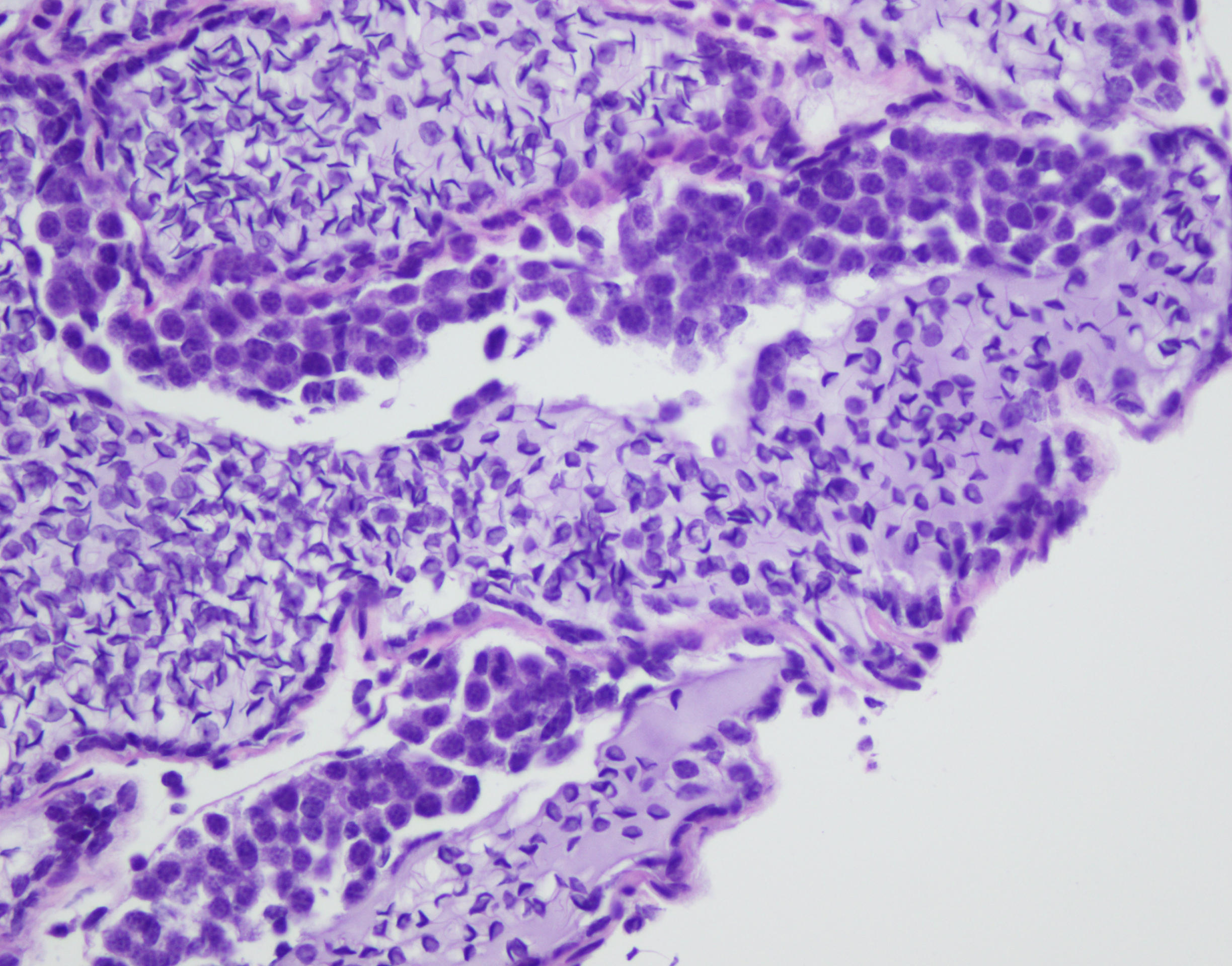

Supplement: Supplementary file 3 — Supplementary Figure 3. [file 41598_2021_99022_MOESM3_ESM.jpg]

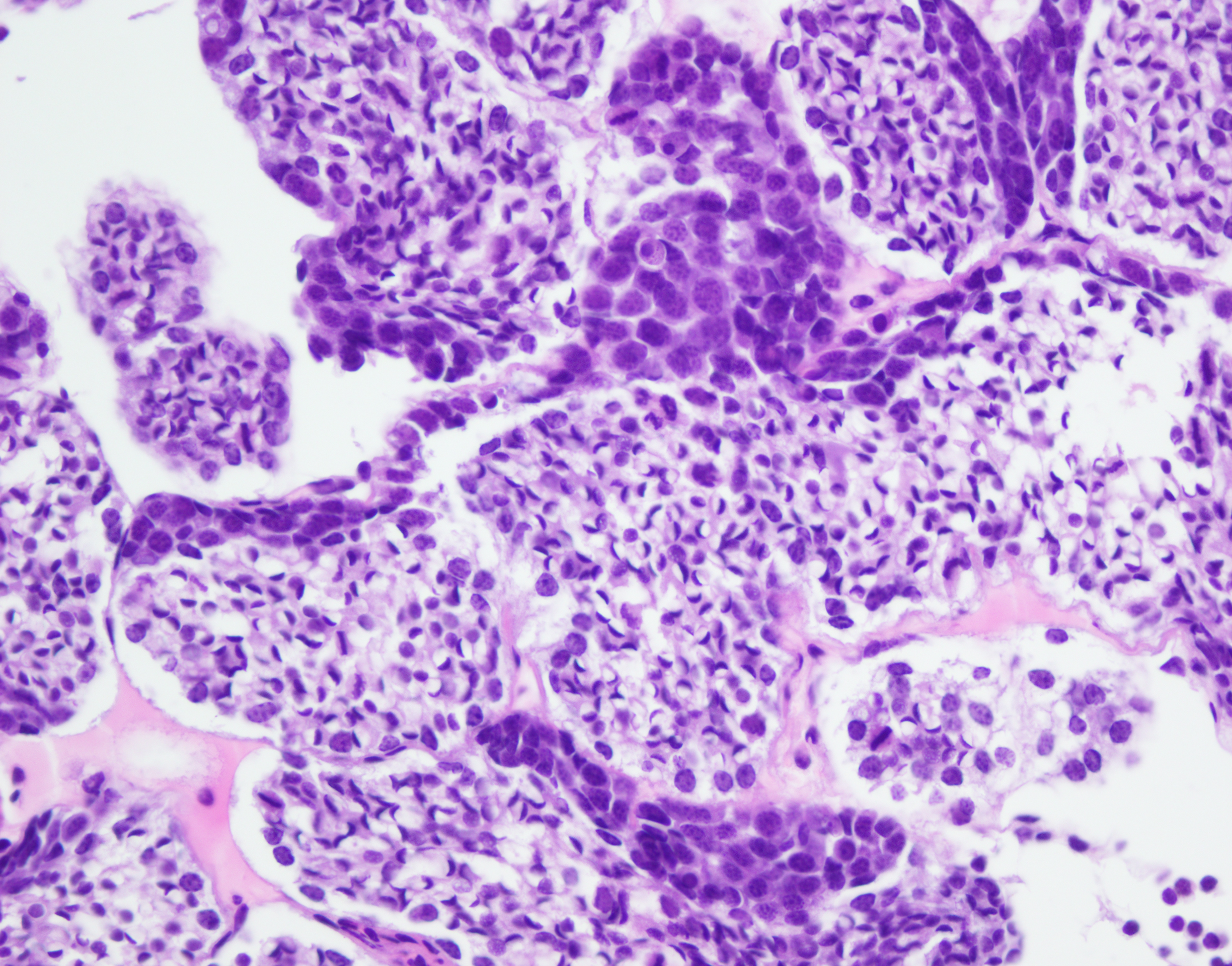

Supplement: Supplementary file 4 — Supplementary Figure 4. [file 41598_2021_99022_MOESM4_ESM.jpg]

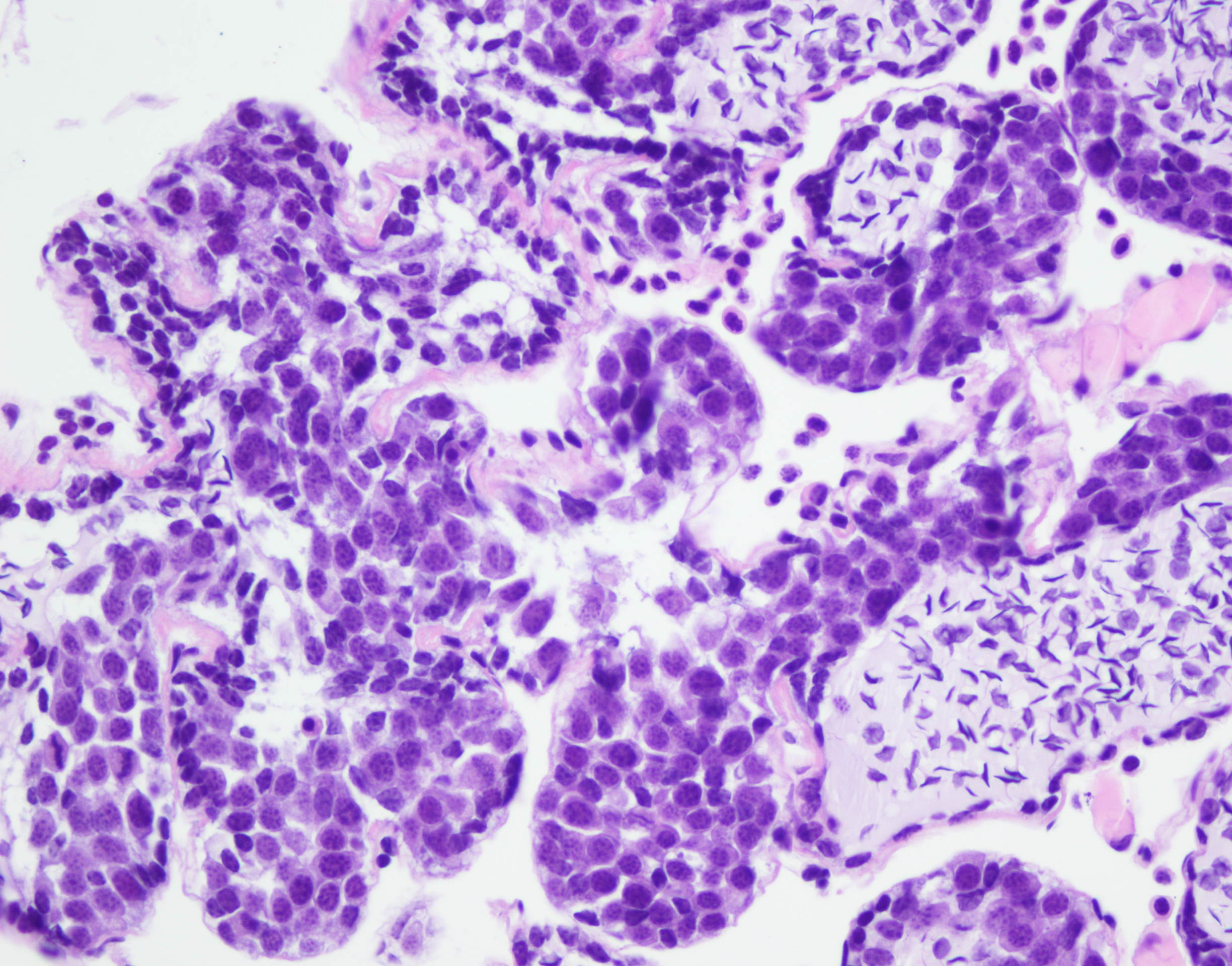

Supplement: Supplementary file 5 — Supplementary Figure 5. [file 41598_2021_99022_MOESM5_ESM.jpg]

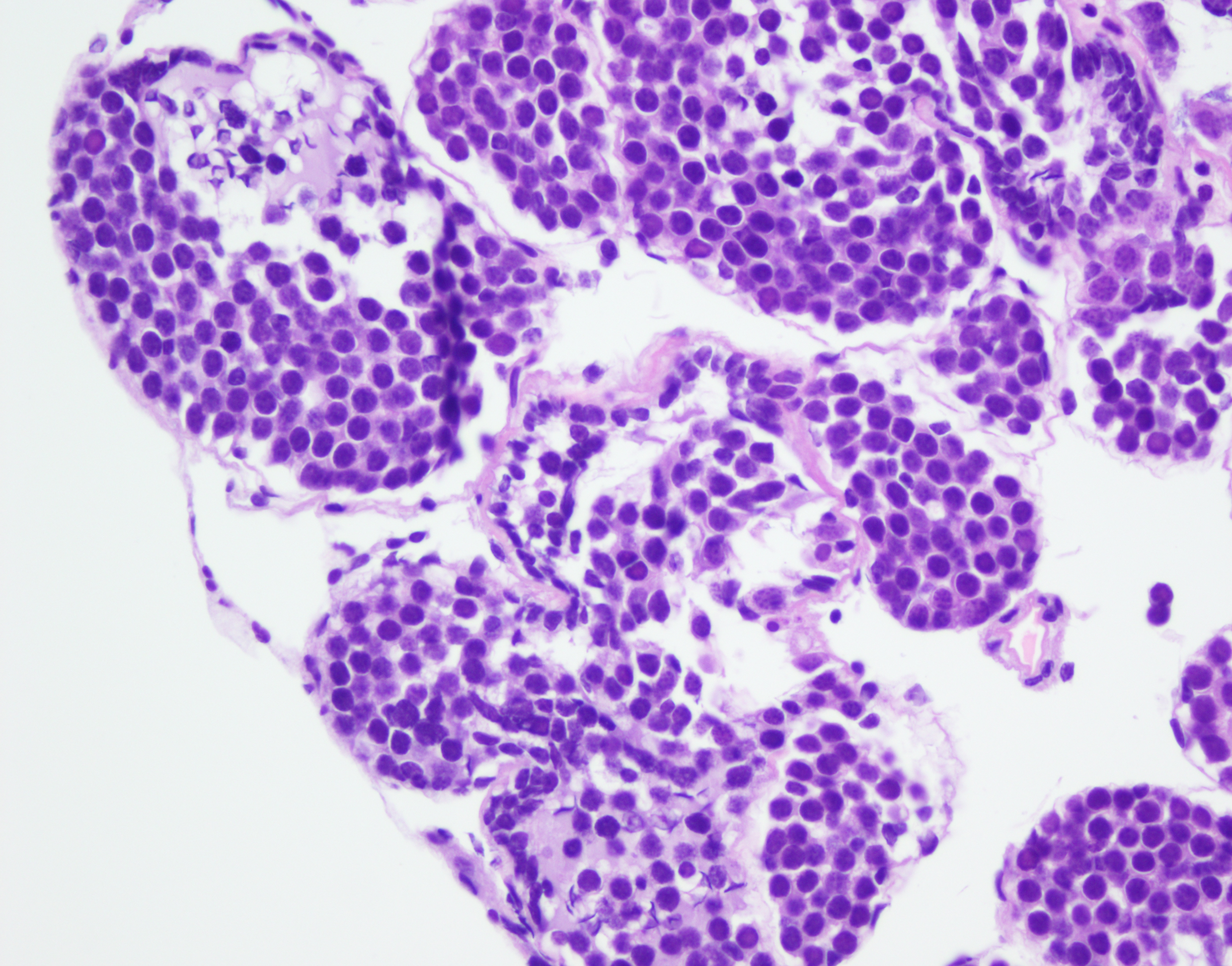

Supplement: Supplementary file 6 — Supplementary Figure 6. [file 41598_2021_99022_MOESM6_ESM.jpg]
